# Low Molecular Weight Heparin Improves the Inflammatory State of Acute Sinusitis Rats Through Inhibiting the TLR4-MyD88-NF-κB Signaling Pathway

**DOI:** 10.3389/fphar.2021.726630

**Published:** 2021-11-17

**Authors:** Tong Wu, Sihan He, Zan Jiao, Xiang Liang, Yu Chen, Huow Liu, Yongq Zhang, GuangX He

**Affiliations:** ^1^ Department of Otolaryngology-Head Neck Surgery, Third Xiangya Hospital, Central South University, Changsha, China; ^2^ Department of Head and Neck Surgery, State Key Laboratory of Oncology in South China, Collaborative Innovation Center of Cancer Medicine, Sun Yat-sen University Cancer Center, Guangzhou, China; ^3^ Institute of Molecular Precision Medicine, Xiangya Hospital and Center for Medical Genetics, Central South University, Changsha, China

**Keywords:** low molecular weight heparin, natural sulfated glycosaminoglycan, acute sinusitis, anti-inflammation, nuclear factor kappa-B

## Abstract

**Introduction:** Low molecular weight heparin (LMWH), a natural sulfated glycosaminoglycan with an affinity for proangiogenic factors, is produced by chemical or enzymatic depolymerization of unfractionated heparin (UFH). Known for its anticoagulant effects, LMWH has recently been reported to have a strong anti-inflammatory effect on colitis, myocarditis, and airway inflammation. However, as a newly-developed drug, its anti-inflammatory mechanism in upper respiratory tract inflammation has not been well-studied.

**Methods:** SD rats were randomly divided into control and experimental groups. The experimental group was established by building an acute nasal sinusitis model with expansion sponges mixed with *Streptococcus pneumoniae*. Then the experimental group rats were subcutaneously injected with different concentrations of LMWH. After seven consecutive days of injection, some rats were sacrificed, and blood and nasal mucosa samples were taken to determine their inflammation status. The remaining acute sinusitis rats were randomly selected for a week of nasal irrigation with normal saline or saline mixed with different concentrations of LMWH. One week later, rats were sacrificed, and samples of blood and nasal mucosa were taken to determine the inflammation status.

**Results:** Rat nasal mucosa in the model group had obvious inflammation. The degree of nasal mucosa inflammation damage in the experimental group was lower than in the experimental control group, proving that LMWH has a protective effect on the nasal mucosa and that the effect correlates with dosage. Irrigation of the nose with saline mixed with LMWH can improve the anti-inflammatory effect. Protein related to the TLR4-MyD88-NF-κB signaling pathway was activated in the acute sinusitis rat model, and LMWH can significantly inhibit its expression.

**Conclusion:** This is the first report of the anti-inflammatory effect of LMWH in acute upper respiratory tract inflammation, together with an explanation of its anti-inflammatory mechanism. The findings contribute a theoretical basis for its potential anti-tumor effect.

## Introduction

Heparin has advantages in treating sepsis due to its anti-inflammatory and anticoagulant effects (Cornet et al., 2007; Zarychanski et al., 2015). It can be divided into unfractionated heparin (UFH) and low molecular weight heparin (LMWH) (Fan et al., 2016). LMWH is produced by chemical or enzymatic depolymerization of UFH (Yan et al., 2017; Yu et al., 2019). It is a promising heparin derivative because of its superior antithrombotic effect, better bioavailability, and lower risk of bleeding (Fan et al., 2016). Fries et al. found that LMWH can interfere with the tissue infiltration of inflammatory cells. Thus, the rolling, adhesion, and tissue infiltration of white blood cells on endothelial cells will also be inhibited and have an anti-inflammatory role (Fries et al., 1998). Wan investigated 12 patients diagnosed with colitis and found that LMWH could inhibit leukocyte accumulation (Wan et al., 2001). Pan found that a non-anticoagulant species was generated from partial desulfation of LMWH to fully retain the anti-inflammatory activity (Pan et al., 2020). LMWH has been reported to have a strong anti-inflammatory effect in various kinds of inflammation, such as colitis (Ahmad et al., 2021), myocarditis (Frizelle et al., 1992), and airway inflammation (Coyle et al., 1994). However, the anti-inflammatory effect of LMWH and its mechanism on the upper respiratory tract, such as in acute sinusitis, has not been studied.

Acute sinusitis, a symptomatic inflammation of the paranasal sinuses and nasal cavity, is reported annually in nearly 30-million adults (Blackwell et al., 2014). Although upper respiratory tract viruses cause most episodes of acute sinusitis, they are also associated with asthma, allergic rhinitis, smoking, and exposure to second-hand smoke (Berrettini et al., 1999; Brook and Hausfeld, 2011; Tan and Corren, 2011; Hur et al., 2014). Medications for acute sinusitis include antibiotics, glucocorticoids, mucosal drainage enhancers, and decongestants. However, in clinical management, unreasonable or abusive use of drugs often leads to problems such as bacterial resistance and recurrence. Therefore, new methods are urgently needed to improve the treatment effect of acute and chronic rhinosinusitis, alleviate patients’ symptoms and improve their quality of life.

Toll-like receptors (TLRs) belong to pattern recognition receptors (PRRs), the first described member of the TLR family. They mediate the inflammatory response in the myocardium. In addition, their mediation of the inflammatory signaling pathway plays a key role in inflammation and ischemia-reperfusion injury (Lu et al., 2015). Multiple studies have demonstrated that TLR4-MyD88-NF-κB signaling controls the production of pro-inflammatory factors and induces the inflammatory response (Zhang et al., 2017). Babazada found that NAHNP, a derivative of heparin, significantly inhibited lipopolysaccharide-induced activation of the TLR4-MyD88 signaling pathway and the production of pro-inflammatory cytokines such as TNF-*α* from mouse macrophages (Babazada et al., 2014). LMWH has a similar molecular structure to NAHNP, but whether LMWH can suppress the TLR4-MyD88-NF-κB pathway and has an anti-inflammatory role needs to be studied.

Based on this background, LMWH appears suitable for treating acute sinusitis by inhibiting the TLR4-MyD88-NF-κB pathway. Therefore, this paper is premised on the hypothesis that LMWH could play an anti-inflammatory role in treating acute sinusitis and protect the nasal mucosa by inhibiting the TLR4-MyD88-NF-κB signaling pathway. In the present study, rats were used to study the effect of LMWH on acute sinusitis and its related molecular mechanisms.

## Methods

### Experimental Animals and Treatment Methods

All rats used were specific pathogen-free (SPF) grade adult male rats, weighing about 300 g and purchased and used based on protocols approved by the Animal Care and Use Committee of the Third Xiangya Hospital of Central South University. They were kept and fed in a pathogen-free environment, and did not have diseases of the nasal cavity or upper respiratory tract. Each group of animals was isolated in a biohazard containment facility. Four rats were included in the blank control group. Rats in the model group were divided into control group, low-dose LMWH group (75 u/kg), medium-dose LMWH group (150 u/kg), and high-dose LMWH group (300 u/kg) according to the concentration of injected LMWH. Six rats were included in each group. The rats were medicated for 7 days. Then they were all sacrificed, and samples of blood, nasal mucosa and nasal flushing fluid were taken. Supplementary Figure S1 provides an overview of the experimental process.

### Building the Acute Sinusitis Model

*Streptococcus pneumoniae* (ATCC49619), previously proved to effectively induce an acute sinusitis model, was used to induce acute sinusitis. It was grown on blood agar plates, and colonies were suspended in sterile saline solution immediately before injection of the rats. Rats were anesthetized with 10% chloral hydrate solution according to the standard injection dose of 3 ml/kg–4 ml/kg by abdominal subcutaneous injection. Their noses were filled with an expansion sponge, small droplets of bacterial solution were placed onto the external nares, and the fluid was drawn into the nasal passages through the expansion sponge for 7 days.

### Sample Collection

Rats were anesthetized with chloral hydrate, and blood was obtained from the orbital venous sinus. Then samples were spun at 5,000 rpm, and the sera were collected and stored at −20°C until assayed. After the nasal bone was removed, the whole nasal mucosa was carefully extracted and fixed in 10% neutral-buffered formalin for the next assay.

### Nasal Bacterial Culture

The rats were anesthetized and lavaged with 300 μL phosphate-buffered saline (PBS). Then the recovered lavage solution was plated on blood agar plates of Columbia sheep after a 100-fold dilution. The plates were incubated for 48 h, and then *Streptococcus pneumoniae* colonies were counted. The colony formation unit (CFU) was used to estimate the number of bacterial colonies, and data were recorded as CFU per microliter of sino-nasal lavage samples. Lavage cultures correlated (*r* = 0.848; *p <* 10^−6^) with cultures of ground sinus tissue (Jin et al., 2011).

### H&E Staining

Fixed skulls were decalcified in Surgipath Decalcifier II (Surgipath, Richmond, IL, United States) and washed three times in PBS. The tissue was rapidly dehydrated at 48°C by a series of ethanol and xylene, then infiltrated with low-temperature paraffin under vacuum pressure. The nasal cavity was cut at 5 μm with a slicer and then mounted on glass slides. The sections were stained with hematoxylin and eosin. A single section was analyzed by a computer-aided optical microscope combined with reconstruction and imaging software (Neurolucida, Microbrightfield, VT, United States).

### Enzyme-Linked Immunosorbent Assay (ELISA)

The levels of IL-6 and TNF-*α* in serum samples and cell supernatants were examined with specific ELISA kits (R&D Systems, Minneapolis, MN, United States) according to the manufacturer’s instructions. Sample concentrations were read from a calibration curve.

### Western Blot

SDS-PAGE was electro-transferred onto PVDF membranes (Millipore, Atlanta, GA, United States). The membranes were blocked with 5% bovine serum albumin or nonfat milk for 1.5 h at room temperature and incubated at 4°C overnight with primary antibodies against TLR4, MyD88, P65, p-P65, or GAPDH. Primary antibodies specific to TLR4 were supplied by Abcam Biotech (Cambridge, United Kingdom). Primary antibodies targeting MyD88, P65, and p-P65 were obtained from Cell Signaling Technology (Beverly, MA, United States), and diluted to 1:1000 according to the instructions. After washing 5 times with tris-buffered saline (TBS) containing 0.1% Tween 20 (TBST), the membranes were incubated with secondary antibodies conjugated with horseradish peroxidase in TBST for 2 h at room temperature. Immunoreactive bands were detected with an enhanced chemiluminescent detection system (Pierce, Rockford, IL, United States), and protein amounts were quantified with Image Lab software (Bio-Rad).

### RNA Extraction and Quantitative Real-Time PCR (RT-qPCR)

Total RNA was extracted using the Trizol reagent (Invitrogen, Carlsbad, CA, United States). NanoDrop (Thermo Fisher Scientific Inc., United States) was then used for reverse transcription using a cDNA reverse transcription kit (TaKaRa, Japan) according to the manufacturer’s instructions. Then, the obtained cDNA was subjected to RT-qPCR for TLR4 mRNA using an SYBR Green I PCR kit (TaKaRa, Japan). All RT-qPCR reactions were performed on the ABI PRISM 7500 system (Applied BioSystems, United States). Glyceraldehyde-3-phosphate dehydrogenase (GAPDH) was used as an internal control. The relative quantification of mRNA expression was calculated using the 2^−ΔΔCt^ method and normalized to GAPDH.

The sequences of the primers were designed as follows:

TLR4 forward: 5′-AAG​TTA​TTG​TGG​TGG​TGT​CTA​G-3′

reverse: 5′-GGT​TGT​TTC​TGC​TAA​G-3′.

MyD88 forward: 5′- CGA​GAG​CTG​GAG​CAA​ACG​GAG​TTC​AAG-3′

reverse: 5′-GCT​GGC​TAG​TGA​TGG​ACC​ACA​CGC​A-3′.

P65 forward: 5′- AGC​GAG​GCA​TTA​GTG​AGA​TTG-3′

reverse: 5′-GTC​GGT​TTC​GTG​AAG​GAG​ATT -3′.

GAPDH forward: 5′-TGC​ACC​ACC​AAC​TGC​TTA​G-3′

reverse: 5′-GAT​GCA​GGG​ATG​ATG​TTC-3′.

IL-6 forward: 5′-GGC​CCT​TGC​TTT​CTC​TTC​G-3′

reverse: 5′-ATA​ATA​AAG​TTT​TGA​TTA​TGT-3′.

TNF-*α* forward: 5′-CAG​CCT​CTT​CTC​CTT​CCT​GA-3′

reverse: 5′-GGA​AGA​CCC​CTC​CCA​GAT​AGA-3′.

### Data Analysis

The data were assessed using SPSS 26.0 software (IBM, Chicago, IL, United States). All experiments were performed in triplicate, and Student’s t-test was used to analyze the significance of the levels between the two groups.

## Results

### Filling the Nasal Cavity of Rats With an Expansion Sponge Mixed With *Streptococcus pneumoniae* is an Effective Method to Establish a Model of Acute Sinusitis

**FIGURE 1:**
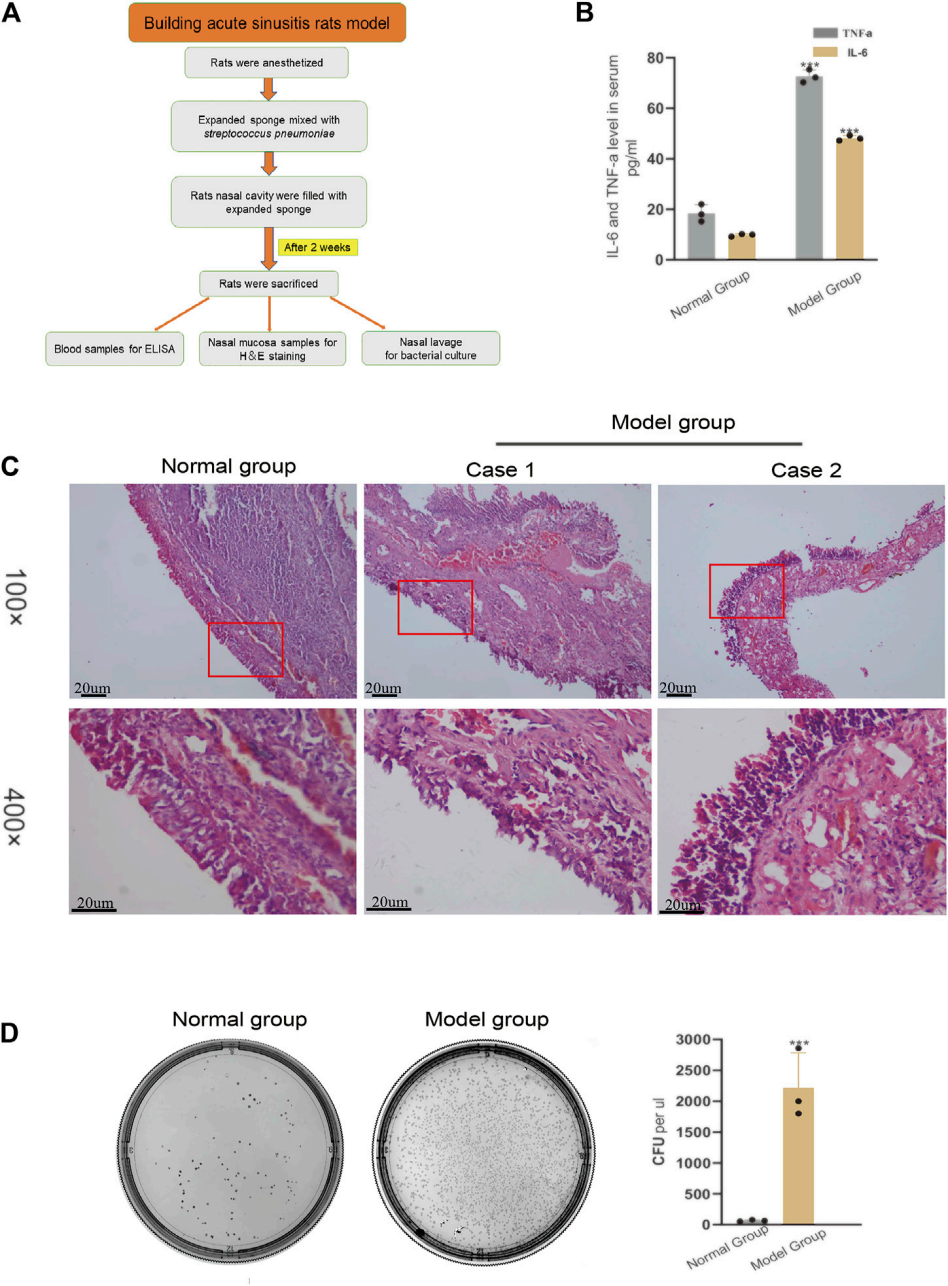
Verifying the successful modeling of acute sinusitis in rats. **(A)** The basic flow chart of the experiment. **(B)** The relative expression of inflammatory cytokines TNF-*α* and IL-6 in the model group and the normal group rats by ELISA. **(C)** Comparison of H&E staining of nasal mucosa of rats in the model and normal groups. **(D)** Bacterial culture of nasal mucosal lavage fluid of rats after 3 days. The left shows normal group rats and the right, model group rats. The experiment was repeated three times.

The method of building the rat model of acute sinusitis is briefly described in Figure 1A. Symptoms of acute sinusitis such as scratching the nose, runny nose, drowsiness, and buccal respiration began to appear in the model group with nasal cavity expansion sponge mixed with *Streptococcus pneumoniae* after 3 days. Serum ELISA results showed that the model group had a significant increase in inflammatory factors such as IL-6 and TNF-*α* compared with normal rats (Figure 1B). HE staining showed acute inflammatory changes, such as lack of mucosa, cilia, and epithelial cells, and many inflammatory cells (Figure 1C). We cultured several nasal resident bacteria from nasal secretions in normal group rats such as *S. epidermidis* and *S. aureus*. *Streptococcus pneumoniae* was cultured from nasal secretions of rats in the model group and rapidly grew. The number of bacterial colonies in the model group was significantly higher than the normal rats (Figure 1D). In summary, it seems that filling the nasal cavity of rats with an expansion sponge mixed with *Streptococcus pneumoniae* is an effective method to establish an acute sinusitis model.

### Low Molecular Weight Heparin can Effectively Decrease the State of Inflammation of Nasal Sinusitis

**FIGURE 2:**
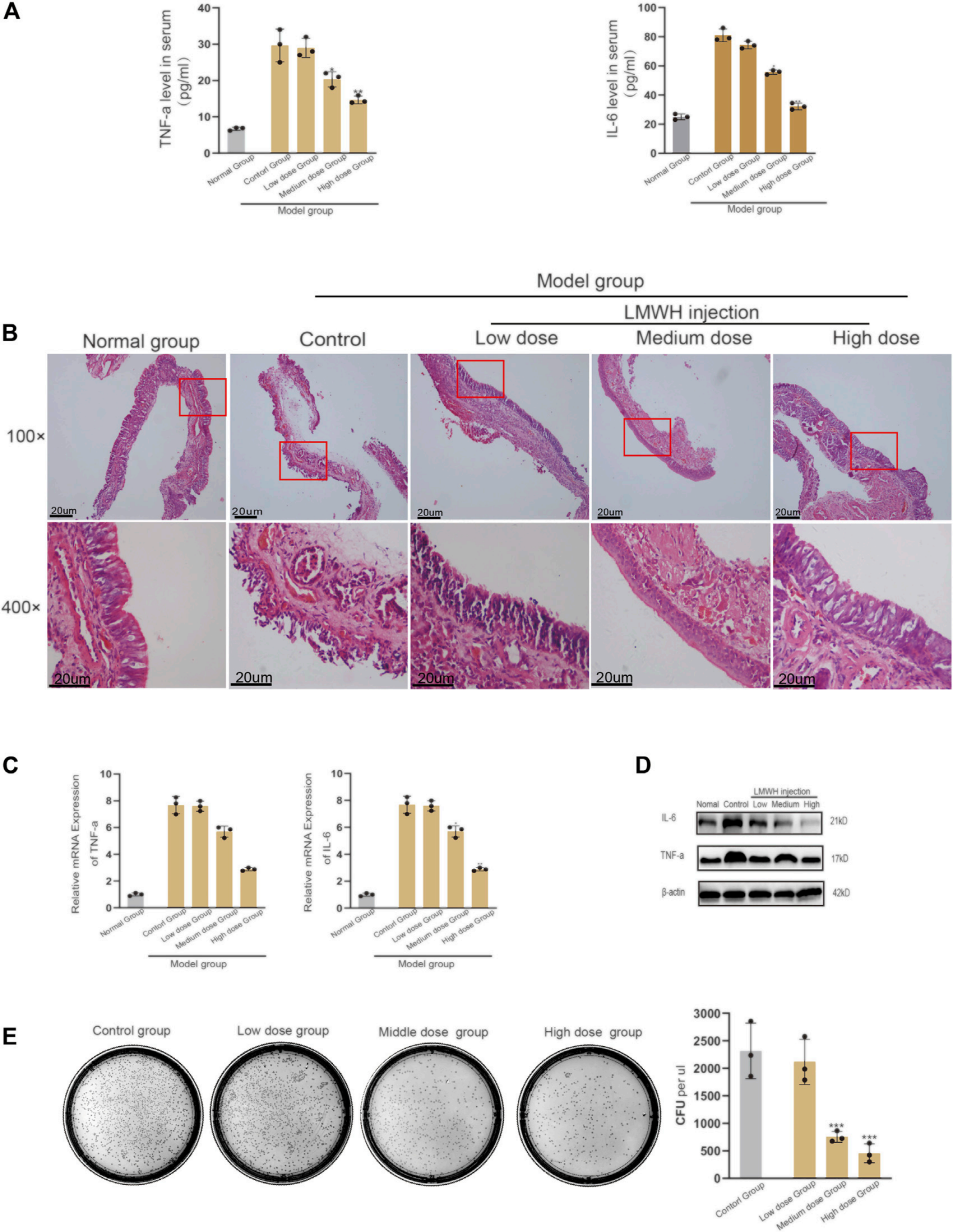
Effect of abdominal injection of LWMH on acute sinusitis in rats. **(A)** Comparison of the relative concentrations of TNF-*α* and IL-6 in the serum of rats after abdominal injection of LMWH at different concentrations. **(B)** H&E staining results of nasal mucosa of rats in each group after abdominal injection of LMWH at different concentrations. **(C)** mRNA level of TNF-*α* and IL-6 was detected by quantitative RT-PCR. **(D)** Protein expression levels of rats after abdominal injection of LMWH at different concentrations by Western blot. **(E)** Bacterial culture and comparison of colony number of sino-nasal lavage samples of rats in each group. Note: Low dose = 75 u/kg, Medium dose = 150 u/kg, High dose = 300 u/kg. ***compared with control group *p* < 0.001. The experiment was repeated three times.

The inflammatory state of rats injected with LMWH was significantly better than that of rats without injection. The serum levels of IL-4 and TNF-*α* decreased with the increase in LMWH concentration (Figure 2A). When the concentration of LMWH reached 150 u/kg and 300 u/kg, the inflammatory factors significantly decreased. The state of nasal mucosa cells showed the same trend. With the increase in LMWH concentration, the inflammatory state of the nasal mucosa in rats was significantly improved, and the number of inflammatory cells and cilia loss decreased (Figure 2B). We then studied the transcription and translation levels. The mRNA expression and protein levels of IL-6 and TNF-*α* in rats injected with LMWH were significantly less than that of rats not injected with LMWH (Figures 2C,D). The relative western-blot quantization results are shown in Supplementary Figures S2A,B. The number of bacterial colonies in the 150 u/kg and 300 u/kg LMWH groups was significantly less than that in rats not injected with LMWH. The degree of decrease was positively correlated with the dose of LMWH (Figure 2E). The symptoms of rats injected with LMWH, such as mouth breathing, sneezing, and runny nose, were much milder than those in the normal group rats. From these results, we consider that LMWH can effectively decrease the state of inflammation of acute sinusitis and can function in a dose-dependent manner.

### Nasal Irrigation Using Normal Saline Mixed With High-Dose Low Molecular Weight Heparin can Improve the Anti-inflammatory Effect of Normal Saline in Acute Sinusitis Rats

**FIGURE 3:**
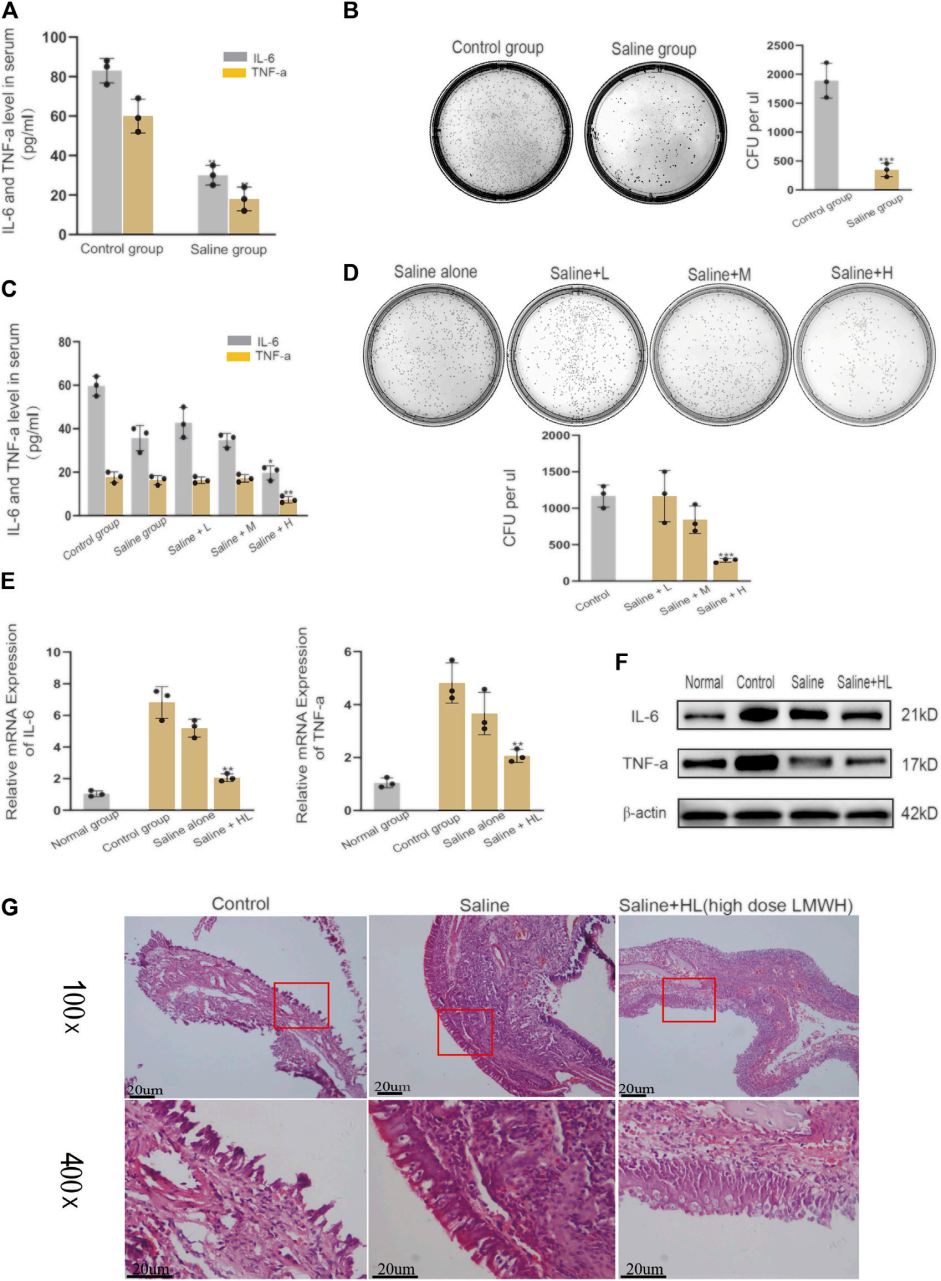
Effect of nasal irrigation using saline mixed with LWMH on acute sinusitis in rats. **(A)** Serum concentrations of TNF-*α* and IL-6 in the normal rats (with no treatment) and the saline group rats (nose cavity was irrigated with saline) were detected by ELISA. **(B)** Bacterial culture and comparison of colony number of sino-nasal lavage sample in the experimental control group and saline groups (nose cavity was irrigated with saline). **(C)** Serum concentrations of TNF-*α* and IL-6 in the saline group rats (nose cavity was irrigated with saline) and the LMWH group rats (nose cavity was irrigated with saline mixed with different concentrations) were detected by ELISA. **(D)** Bacterial culture and comparison of colony number of sino-nasal lavage samples in the saline group and different concentrations of the LMWH group. **(E)** mRNA level of TNF-*α* and IL-6 of each group was detected by quantitative RT-PCR. **(F)** Protein expression levels of rats of each group were determined by Western blot. **(G)** H&E staining results of nasal mucosa of rats in each group. Note: Saline + L = Saline + low dose (75 u/kg) LMWH, Saline + M = Saline + Medium dose (150 u/kg) LMWH, Saline + H=Saline + high dose (300 u/kg) LMWH. *compared with control group *p* < 0.05; **compared with control group *p* < 0.01; ***compared with control group *p* < 0.001. The experiment was repeated three times.

Nasal irrigation with normal saline is an important part of the treatment of acute sinusitis. The results of our experiments showed that LMWH abdominal injection has a strong anti-inflammatory effect. Based on this finding, we continued to explore whether normal saline mixed with varying concentrations of LMWH could enhance the anti-inflammatory effect of normal saline. The acute sinusitis model rats were divided into five groups. One group was irrigated with saline, and three groups were irrigated with saline plus different concentrations of LMWH (75 u/kg, 150 u/kg, and 300 u/kg). One group was given no treatment and served as the control. Comparison of the concentration of inflammatory factors in the blood and the number of cultured colonies in nasal lavage fluid showed that nasal irrigation with saline could significantly improve the inflammatory state of acute sinusitis compared with the control group (Figures 3A,B). At the same time, there was no difference in the anti-inflammatory effect between saline mixed with low and medium concentrations of LMWH and saline alone. However, when saline was combined with a high concentration of LMWH (300 u/kg), the anti-inflammatory effect was significantly stronger than that of saline alone (Figures 3C,D). The results of PCR and Western blot showed that the inflammatory factors such as IL-6 and TNF-*α* were significantly inhibited at both mRNA and protein levels when saline was mixed with a high concentration of LMWH (Figures 3E,F). The relative western-blot quantization results are shown in Supplementary Figures S3A,B. The results of H&E staining also supported this conclusion. When saline was combined with a high concentration of LMWH, the degree of mucosal cilia damage was the least, as was the number of subcutaneous inflammatory cells (Figure 3G). Nasal irrigation using normal saline mixed with high-dose LMWH (300 u/kg) can improve the anti-inflammatory effect of normal saline in acute sinusitis rats.

### Low Molecular Weight Heparin Inhibits Inflammation by Suppressing the Activation of the TLR4-MYD88-NF-κB Signaling Pathway

**FIGURE 4:**
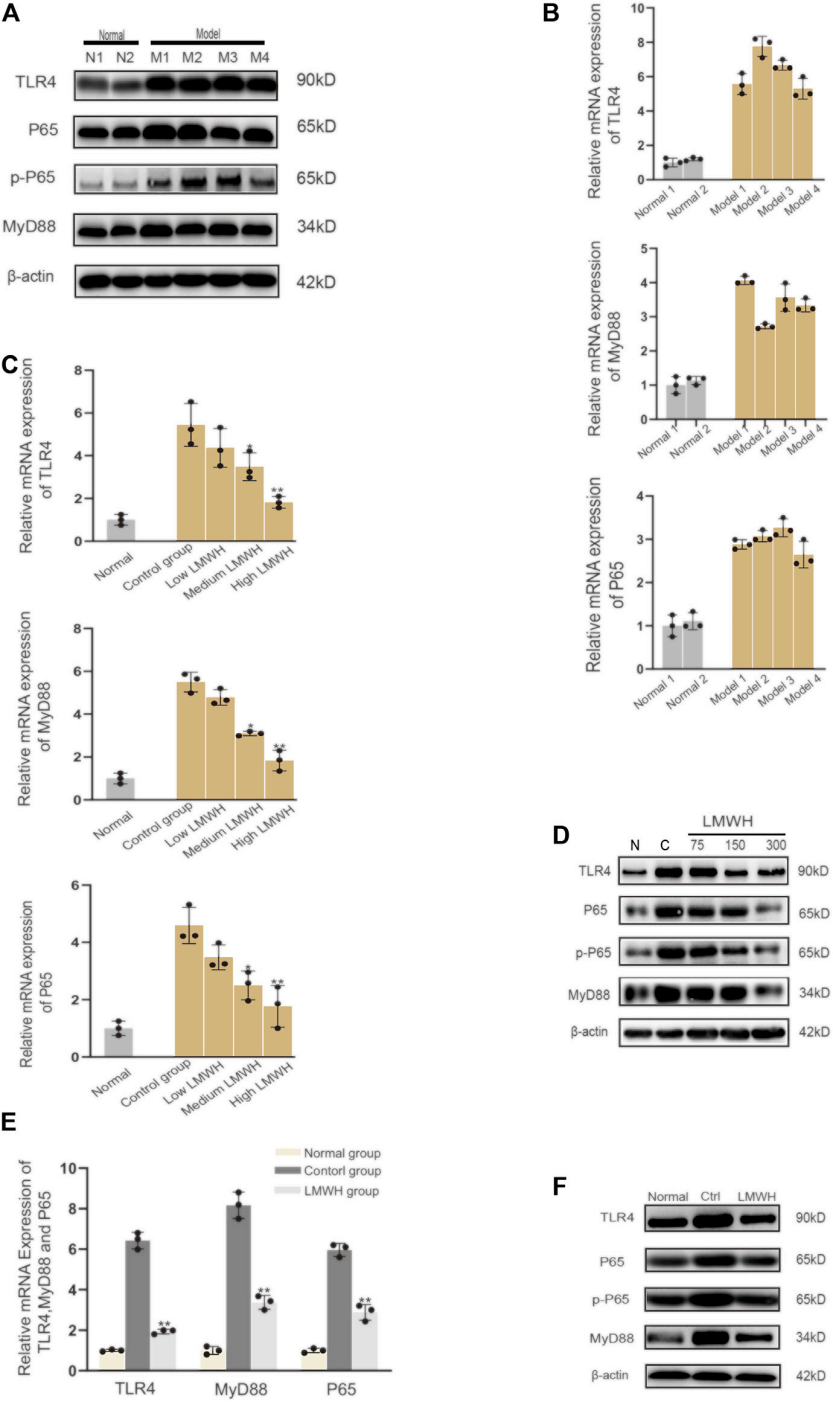
LMWH suppressed the activation of the TLR4-MYD88-NF-κB signaling pathway. **(A)** Expression of TLR4-MYD88-NF-κB pathway-related proteins in normal rats and model rats by Western blot. **(B)** Relative mRNA level of the TLR4-MYD88-NF-κB pathway-related genes in normal rats and model rats by RT-qPCR. **(C)** Relative mRNA level of TLR4-MYD88-NF-κB pathway-related genes in normal rats, experimental control rats, and rats injected subcutaneously with different concentrations of LMWH by RT-QPCR. **(D)** Expression of TLR4-MYD88-NF-κB pathway-related proteins in the normal rats, experimental control group rats, and rats injected subcutaneously with different concentrations of LMWH by Western blot. **(E)** Relative mRNA level of TLR4-MYD88-NF-κB pathway-related genes in normal rats, experimental control group rats, and rats whose nose cavity was irrigated with saline mixed with high dose LMWH by RT-QPCR. **(F)** Expression of TLR4-MYD88-NF-κB pathway-related proteins in normal rats, experimental control rats, and rats whose nose cavity was irrigated with saline mixed with high dose LMWH (300 u/kg) by Western blot. Note: *compared with control group *p* < 0.05. **compared with control group *p* < 0.01; The experiment was repeated three times.

**FIGURE 5:**
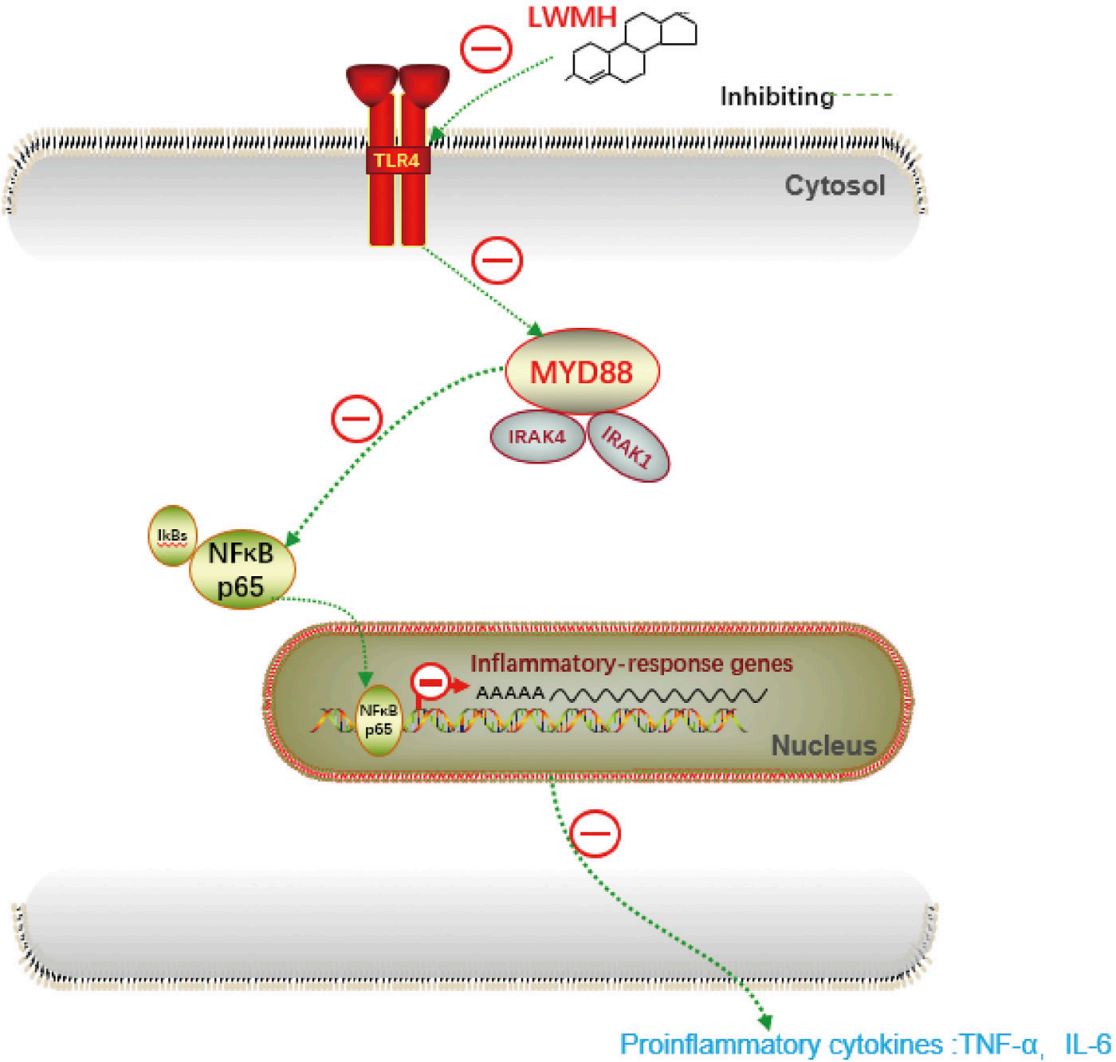
Diagram for inhibition of TLR4-MYD88-NF-κB by LMWH.

The derivatives of heparin NAHNP significantly inhibited lipopolysaccharide-induced activation of the TLR4-MyD88-NF-κB signaling pathway and the production of pro-inflammatory cytokines such as TNF-*α*. LMWH has similar molecular structures to NAHNP, and it was thus, hypothesized that LMWH could also inhibit this pathway. First, we verified that proteins related to the TLR4-MyD88-NF-κB signaling pathway were widely activated in rats with acute sinusitis. Compared with normal rats, the expression of TLR4-MyD88-NF-κB pathway-related genes in mRNA and protein levels of acute sinusitis rats were significantly increased (Figures 4A,B). The relative western-blot quantization results are shown in Supplementary Figure S4A. Then it was found that after abdominal injection of LMWH, the gene expression of related pathways significantly decreased both in mRNA and protein level, and with an increase in LMWH concentration, the decreasing trend was more obvious (Figures 4C,D). The relative western-blot quantization results are shown in Supplementary Figure S4B. Next, the model group was divided into the experimental control group, the saline group, and saline mixed with a high concentration of LMWH (300 u/kg) group. After 7 days of irrigation, PCR and Western blot experiments were carried out on the nasal mucosa. The results showed that both saline and saline mixed with different concentrations of LMWH could significantly inhibit the expression of this pathway, and the inhibition of saline mixed with a high concentration of LMWH (300 u/kg) was higher than that of saline alone (Figures 4E,F). The relative western-blot quantization results are shown in Supplementary Figure S4C. Figure 5 illustrates the inhibition of TLR4-MYD88-NF-κB by LMWH.

In summary, LMWH can inhibit the expression of the TLR4-MyD88-NF-κB pathway and reduce the release of inflammatory factors. Its anti-inflammatory action occurs through both abdominal injection and nasal irrigation.

## Discussion

Acute sinusitis is a common inflammatory disease in Rhinology clinics. However, establishing an acute sinusitis model is very difficult, and the selection of animal models of acute sinusitis has a long history of development. Bomer successfully constructed a model of sinusitis by injecting *Streptococcus pneumoniae* into the nasal cavity of mice (Bomer et al., 1998). Since then, inhalation of *Streptococcus pneumoniae* into the nasal cavity of mice has become a mainstream method to build the acute sinusitis model. A study demonstrated that nasal sponge insertion could induce an acute sinusitis mouse model, but the inflammatory response was less severe than when a nasal sponge impregnated with *Staphylococcus aureus* suspension was inserted (Jin et al., 2011). In our series, we found that inhalation of *Streptococcus pneumoniae* liquid into an expansion sponge and filling the nasal cavity of rats was a very effective way to induce acute sinusitis. The larger size of rats makes it easier to extract nasal mucosa and serum compared with mice. Rats are also more robust than mice and do not easily die during the anesthesia and modeling process. When the nasal cavity is blocked with an expansion sponge, it obstructs ventilation and drainage, but *Streptococcus pneumoniae* accelerates and improves modeling efficiency. Therefore, we achieved effective results with this method.

Cytokines, as key regulators of inflammation, play an important role in the pathophysiology of acute sinusitis. A series of up-regulated cytokines was detected in acute viral rhinitis, including IL-1*β*, IL-6, IL-7, IL-17, IFN-*γ*, TNF-*α*, IL-8, G-CSF, and GM-CSF11 (Klemens et al., 2007). Our data demonstrated that serum levels of IL-6 and TNF-*α* were significantly increased in nasal packing-induced acute sinusitis rats, which were mainly Th1-related cytokines, and consistent with the pathogenic mechanism of acute sinusitis.

LMWH is widely used for the prevention and treatment of thromboembolic disorders. In addition to its well-known anticoagulant effects, interest has grown in its anti-inflammatory effects. Several studies have reported anti-inflammatory effects of LMWH (Table 1) at experimental levels. LMWH shows strong anti-inflammatory effects in inflammatory diseases such as colitis, asthma, and chronic obstructive pulmonary disease. The anti-inflammatory effect of LMWH is linked to its ability to interfere with almost every stage of leukocyte transmigration, largely mediated by IL-6 and TNF-*α* (Pan et al., 2020). In our series, LMWH effectively decreased the release of IL-6 and TNF-*α* when its injection concentration reached 300 u/kg. It seems reasonable that LMWH can greatly reduce the inflammatory state of acute sinusitis, which causes the large-scale release of cytokines IL-6 and TNF-*α*.

**TABLE 1:** Anti-inflammatory effect of LMWH.

Study	Indications	Anti-inflammatory mechanism
current study	acute sinusitis	inhibiting TLR4-MyD88-NF-κB signaling pathway
Shastri et al. (2015)	Asthma	inhibition of T-cell mediated cytokine release
Brown et al. (2006)	chronic obstructive pulmonary disease	anti-inflammation
Dotan et al. (2001), Yalniz et al. (2012)	ulcerative colitis	anti-inflammation
Assy et al. (2007)	liver fibrosis	suppressing expression of inflammatory cytokines
Li et al. (2015)	peritoneal fibrosis	suppressing of inflammatory cytokines
Bergamaschini et al. (2009)	Alzheimer’s disease	Lowering β-amyloid plaque deposition
Luan et al. (2014), Ning et al. (2015)	endotoxin-induced systemic inflammation	Decreasing the expression of Inflammation factors

Although antibiotics are commonly used to control acute respiratory tract infections of bacterial origin, antibiotic resistance is a global problem that hinders the treatment of bacterial infections. To avoid antibiotic resistance, the development of non-antibiotic anti-inflammatory drugs to treat bacterial inflammation may be logical. Glycyrrhetinic acid and hyaluronic acid were reported as non-antibiotic agents effective against acute sinusitis (Ciofalo et al., 2017). In recent years, the anti-inflammatory effect of ethyl pyruvate has attracted extensive attention from scientists, and Liang found that it can ameliorate the inflammatory response of sino-nasal mucosa by inhibiting high mobility group box 1(HMGB1) in rats with acute rhino-sinusitis (Liang et al., 2021). The present study found that LMWH can greatly reduce the release of inflammatory mediators and reduce the formation of bacterial colonies by both subcutaneous injection and nasal irrigation. LMWH can simultaneously fight against inflammatory mediators and inhibit bacterial growth, and its use may help address antibiotic abuse.

The anti-inflammatory effect of LMWH is mediated by its ability to bind and inactivate proteins such as complement components, chemokines, growth factors, tissue-destroying enzymes, and adhesion molecules, such as L- and P-selectin, involved in the recruitment of inflammatory cells (Tyrrell et al., 1999; Wang et al., 1999; Ludwig, 2009). Babazada found that glycol-split heparin nanoparticles inhibit TNF-*α* production in LPS-stimulated macrophages by inhibiting TLR4-mediated NF-κB signaling (Bomer et al., 1998). The interaction of LPS with the TLR4-MD-2 receptor complex leads to activation of the MyD88-dependent downstream signaling pathway. This pathway induces the phosphorylation and degradation of IκB*α*, allowing active NF-κB to enter the nucleus and induce the expression of pro-inflammatory cytokine genes. The recruitment and phosphorylation of IRAK1 are specific to the MyD88-dependent signaling pathway (Li and Verma, 2002). Bhel et al. (Behl et al., 2021) described how LMWH reduced HMGB1 expression during inflammation by negatively mediating NETosis. LMWH could decrease inflammatory factors in diabetes by inhibiting the HMGB1-TLR4-NF-κB pathway, which gave rise to our study of the mechanism of interaction of LMWH and TLR4 in acute sinusitis. The present study found that LMWH can cause the release of inflammatory cytokines such as IL-6 and TNF-*α* by inhibiting the TLR4-MyD88-NF-κB signaling pathway. The inhibitory effect was most obvious when the LMWH concentration was high (300 u/kg). To our knowledge, this is the first time that LMWH has been shown to exhibit enhanced anti-inflammatory properties by acting as a TLR4 antagonist through the TLR4-MyD88 pathway. Yu et al. (Yu et al., 2019) reported that LMWH has a potential therapeutic effect on progressive tumors. However, the mechanism was not clarified in the article. In recent years, activation of the TLR4-MyD88-NF-κB signaling pathway has been linked to cancer progression and chemotherapeutic drug resistance (Kfoury et al., 2013). Given its ability to inhibit the activation of the TLR4-MyD88-NF-κB signaling pathway, the anti-tumor effect of LMWH seems to hold great potential, and its anti-tumor effects deserve further study.

Although our study proved that LMWH suppressed the inflammatory response of nasal sinus mucosa in rats with acute sinusitis, the most suitable concentration and appropriate treatment time remain unclear. Clinical trials suggest LMWHs as promising treatment options for inflammatory disorders. For example, children and diabetic patients undergoing cataract surgery were prescribed enoxaparin to reduce the incidence of inflammatory syndrome (Ilhan et al., 2013). Our study found that LMWH can play a strong anti-inflammatory role in acute sinusitis. It provides a theoretical basis for the clinical application of LMWH in acute sinusitis patients. It is not clear whether LMWH could benefit acute sinusitis patients in attenuating the inflammatory response of the nasal sinus, and clinical trials are necessary to better understand LMWH’s role in patients with acute sinusitis. We suggest that LMWH also shows great potential anti-tumor effects because of its ability to inhibit the TLR4-MyD88-NF-κB signaling pathway, related to cancer progression and chemotherapeutic drug resistance. This anti-tumor effect of LMWH requires further investigation.

## Conclusion

LMWH has potential in the treatment of acute sinusitis. Our study found that LMWH can improve the inflammation state of acute sinusitis and has a dose-dependent protective effect on the nasal mucosa, both through abdominal subcutaneous injection and nasal irrigation in rats. This result suggests further treatment modalities for acute sinusitis and provides new suggestions for preventing antibiotic abuse. The protective effect of LMWH on the nasal mucosa of rats with sinusitis may be related to inhibiting the activation of the TLR4-MyD88-NF-κB signaling pathway and down-regulating the gene transcription of inflammatory factors in a dose-dependent manner, thus reducing the production of inflammatory factors. This is the first time LMWH has been shown to exhibit enhanced anti-inflammatory properties by acting as a TLR4 antagonist through the TLR4-MyD88 pathway. The findings are valuable in further clarifying the anti-inflammatory mechanism of LWMH and have practical implications in providing new possibilities for cancer treatment.

## Data Availability

The raw data supporting the conclusion of this article will be made available by the authors, without undue reservation.

## Supplementary Material

Supplementary Figure S1 Overview of the experimental design.

Supplementary Figure S2 The relative western-blot quantization results of Figure 2.

Supplementary Figure S3 The relative western-blot quantization results of Figure 3.

Supplementary Figure S4 The relative western-blot quantization results of Figure 4.

## Abbreviations

CFU, Colony Formation Unit; COVID-19, 9: Corona Virus Disease 2019; HMGB1, High Mobility Group Box 1; IL-6, Interleukin-6; LMWH, Low Molecular Weight Heparin, LPS:Lipopolysaccharide; MyD88, Myeloid Differentiation Factor88; NAHNP, Non-anticoagulant Heparin/d-erythro-sphingosine Nanoparticle; NF-κB, Nuclear Factor kappa-B; TLR4, Toll-like receptor 4; TNF-a, Tumor Necrosis Factor-a; UFH, Unfractionated Heparin; VILI, Ventilator Induced Lung Injury.
