# Supplementary figures and images for: Low Molecular Weight Heparin Improves the Inflammatory State of Acute Sinusitis Rats Through Inhibiting the TLR4-MyD88-NF-κB Signaling Pathway
Source: Front Pharmacol. 2021 Nov 17;12:726630. doi: 10.3389/fphar.2021.726630 (PMC8635784; doi:10.3389/fphar.2021.726630)

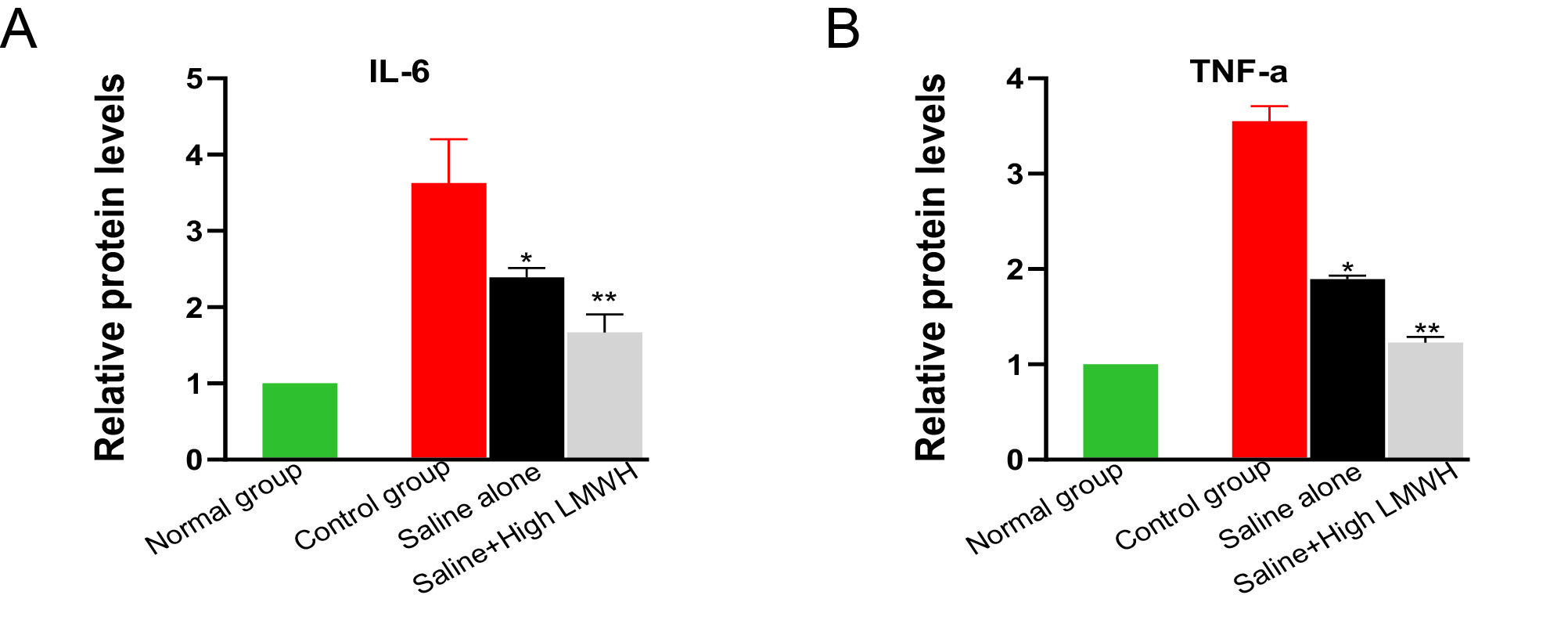

Supplement: Supplementary file 1 [file Image3.JPEG]

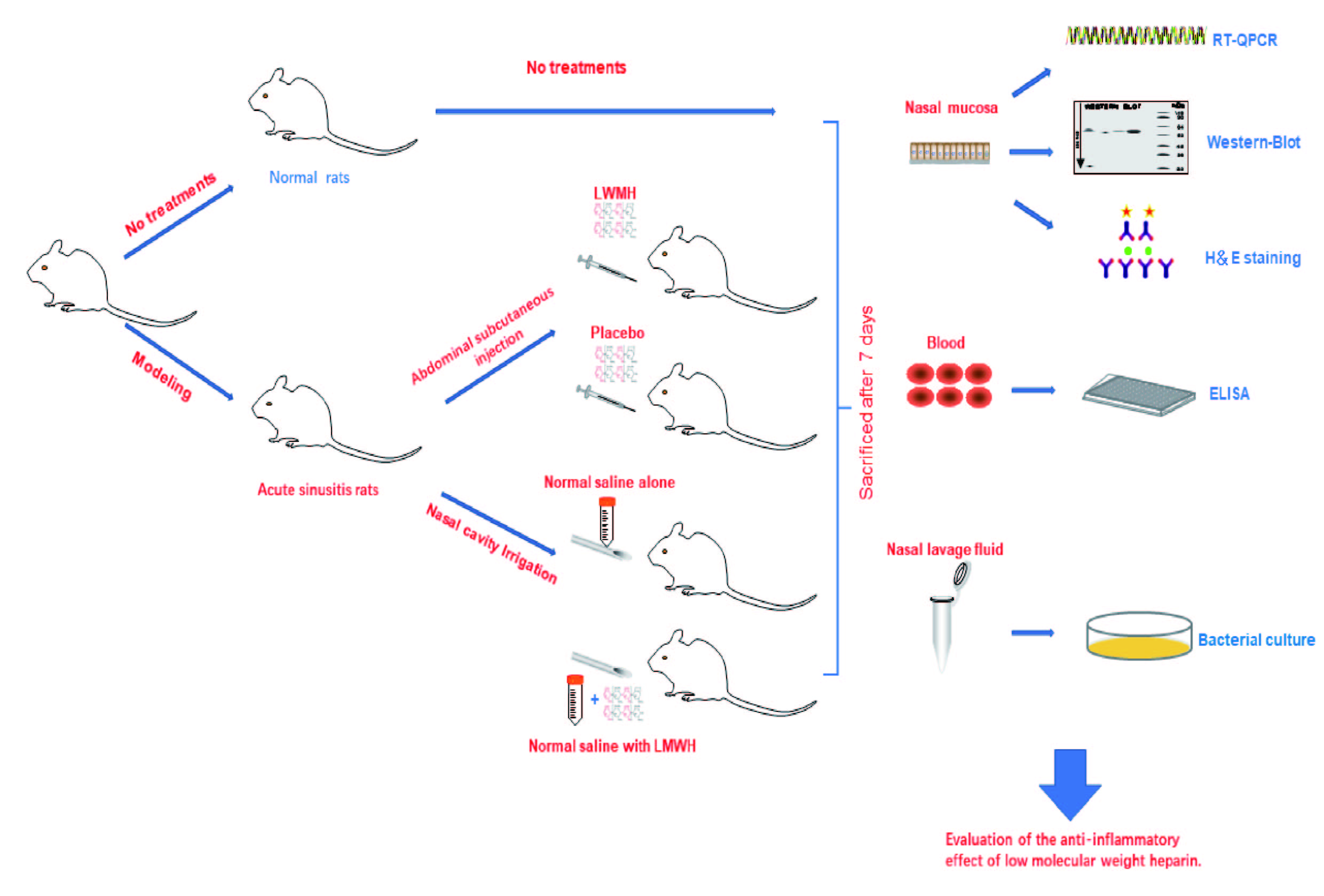

Supplement: Supplementary file 2 [file Image1.JPEG]

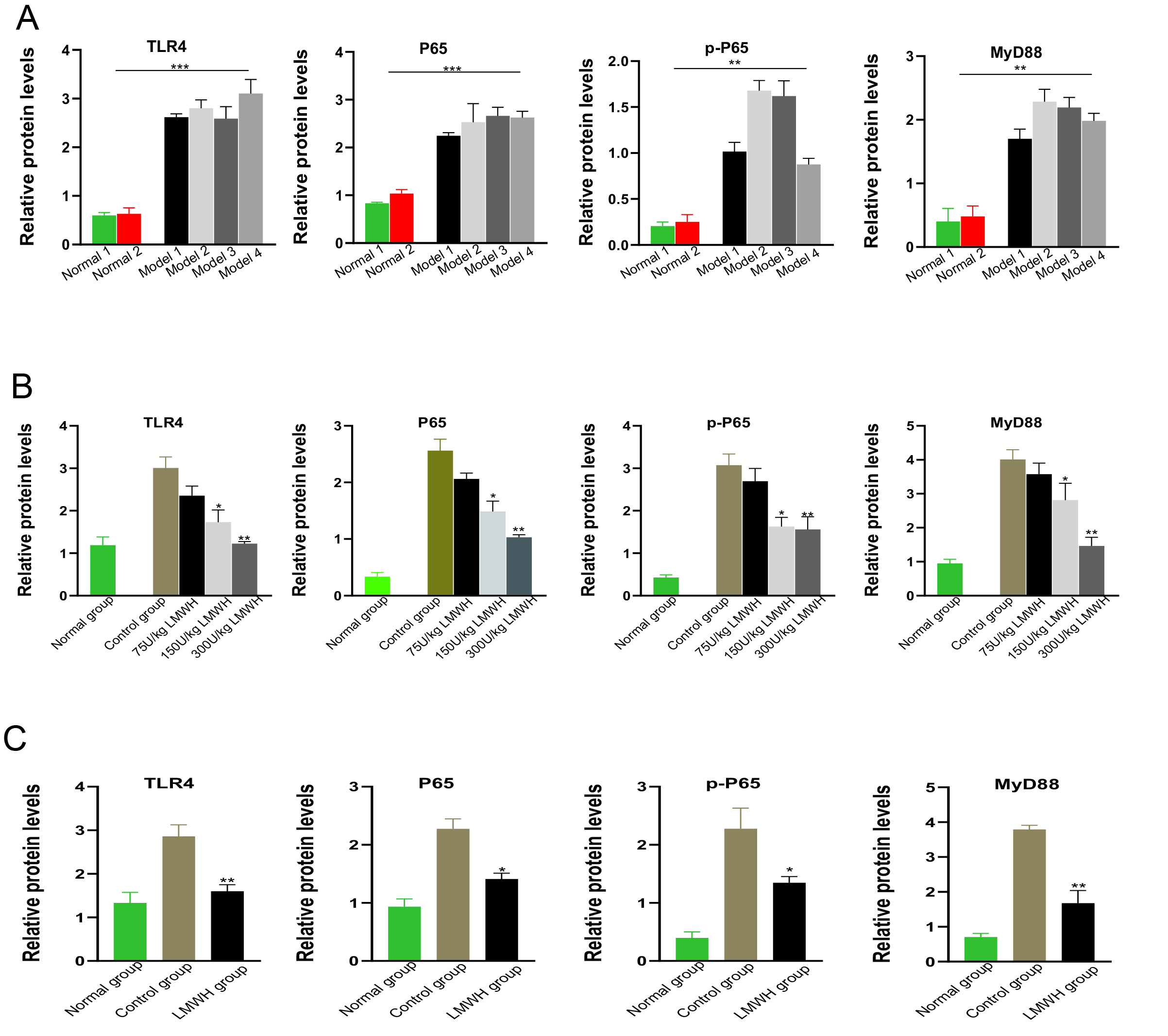

Supplement: Supplementary file 3 [file Image4.JPEG]

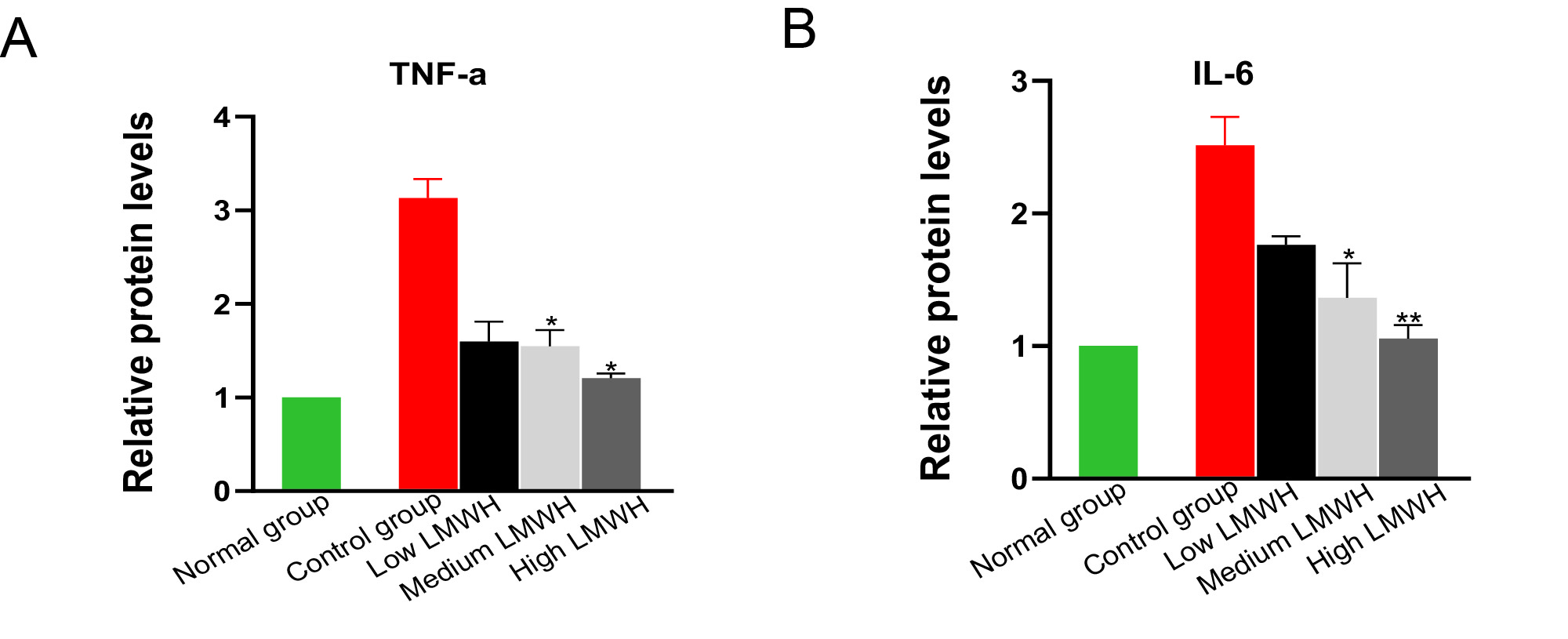

Supplement: Supplementary file 4 [file Image2.JPEG]
